# Adaptation to Arginine Deprivation Leads to a More Aggressive, Therapy-Resistant Phenotype in HNSCC Cells

**DOI:** 10.3390/biom15060900

**Published:** 2025-06-19

**Authors:** Oleg Chen, Olena Vovk, Nikita Polishchuk, Oksana Mayevska, Galyna Shuvayeva, Melike Demir, Vasyl Lukiyanchuk, Leoni A. Kunz-Schughart, Anna Dubrovska, Oleh Stasyk

**Affiliations:** 1 Department of Cell Signaling, Institute of Cell Biology, National Academy of Sciences of Ukraine, Drahomanov Str. 14/16, 79005 Lviv, Ukraine; oleg.chen@lih.lu (O.C.); vovkoliv@gmail.com (O.V.); shuvayeva77@gmail.com (G.S.); 2 Cancer Metabolism Group, Department of Cancer Research, Luxembourg Institute of Health, 1210 Luxembourg, Luxembourg; 3 Ukrainian National Forestry University, General Chuprynky Str. 103, 79057 Lviv, Ukraine; 4 OncoRay-National Center for Radiation Research in Oncology, Faculty of Medicine and University Hospital Carl Gustav Carus, TUD Dresden University of Technology, and Helmholtz-Zentrum Dresden-Rossendorf, 01309 Dresden, Germany; melike.demir@ukdd.de (M.D.);; 5 Helmholtz-Zentrum Dresden-Rossendorf, Institute of Radiooncology-OncoRay, 01328 Dresden, Germany; 6 National Center for Tumor Diseases (NCT), Partner Site Dresden, 01307 Dresden, Germany; 7 German Cancer Research Center (DKFZ), 69120 Heidelberg, Germany; 8 German Cancer Consortium (DKTK), Partner Site Dresden, 01309 Dresden, Germany

**Keywords:** arginine deprivation therapy, recombinant human arginase type 1, head and neck squamous cell carcinoma, acquired drug resistance, epithelial–mesenchymal transition, DNA repair, radiosensitization

## Abstract

*Purpose:* The development of acquired resistance to arginine deprivation therapy (ADT) is a major barrier to its efficacy. This study aimed to elucidate the possible mechanisms underlying the resistance to ADT. *Methods:* We applied repeated ADT and established a subline SAS-R9 of the human head and neck squamous cell carcinoma (HNSCC) cells semi-resistant to arginine (Arg) deprivation *in vitro*. This subline was compared to the parental SAS cell lines for its relative clonogenic proliferation, aggregation, adhesion, and migration capacities. The transcriptomic changes were assessed by RNA sequencing. Signaling pathway alterations were confirmed by RT-PCR and Western blotting. Relative cell radioresistance was analyzed by radiobiological clonogenic survival assay. DNA double-strand break (DSB) repair was assessed by γH2A.X foci analysis. *Results:* SAS-R9 cells showed higher survival in response to ADT and radiotherapy, elevated clonogenic proliferation rate, cell–cell aggregation, and cell–matrix adhesion, along with increased epithelial–mesenchymal transition (EMT) markers and enhanced DNA DSB repair, potentially related to a more aggressive and therapy-resistant phenotype. *Conclusions:* While acute ADT has radiosensitizing potential, this new study suggests that long-term, repeated ADT is associated with cell selection and reprogramming, resulting in resistance to radiotherapy-induced DNA damage and higher tumor cell aggressiveness.

## 1. Introduction

Arginine (Arg), a semi-essential amino acid in humans, has been identified as a key player in numerous processes, including the synthesis of nitric oxide and polyamines, functioning of the cardiovascular and immune systems, as well as a direct activator of mTOR, a nutrient-sensing kinase strongly implicated in carcinogenesis [1]. Some cancer cells are arginine-auxotrophic, showing an inability to sustain growth when deprived of Arg, representing Arg as a druggable target for metabolic anticancer therapy [2,3,4]. These tumors are often heavily reliant on extracellular Arg due to the downregulation of argininosuccinate synthetase (ASS) and/or ornithine transcarbamylase (OTC)—key enzymes for intracellular Arg synthesis [1,5]. Engineered Arg catabolic enzymes (pegylated forms of recombinant human arginase 1 (rhARG1) and bacterial arginine deiminases (ADI)) are currently in preclinical and clinical trials to treat such tumors. One hypothesis for putative treatment failure in these malignancies is the development of resistance due to the upregulation of the initially reduced arginine-anabolic enzymes.

On the other hand, it is considered that even patients with initially non-auxotrophic cancers might benefit from ADT because Arg starvation can lead to sensitization of cancer cells to standard curative-intended treatments such as radiotherapy. In this context, ADT was proposed as a new therapeutic approach for highly aggressive head and neck squamous cell carcinomas (HNSCCs), the seventh most common cancer worldwide, which accounts for 4.5% of all cancer diagnoses and deaths, according to GLOBOCAN estimates [6,7]. New combinatorial treatment options are of utmost interest because the recurrence of the disease after standard therapy and the development of resistance to treatment are significant obstacles in today’s HNSCC patient outcomes, in addition to first diagnosis at late, metastatic stages [8,9,10]. Our previous findings suggest that ADT efficiently sensitizes ASS1-positive HNSCC models, such as SAS cells, to radiation in both two-dimensional (2D) and three-dimensional (3D) assays [11]. It can also efficiently limit 3D growth if applied for longer time periods. However, the risk of acquiring resistance to the metabolic adaptation accompanied by a loss of the radiosensitizing capacity of the approach has yet to be elucidated.

To investigate the phenomenon of resistance development to ADT in ASS1-positive cancer cells and further address the resulting issues, we utilized the initial Arg-non-auxotrophic but sensitive SAS cells to generate ADT-adapted sublines through repeated, controlled exposures to ADT. The subline SAS-R9 finally exhibited an ADT-semi-resistant phenotype and was further characterized in comparison to its ADT-sensitive ancestor cell line SAS. The goal was to identify pathway modulations related to aggressiveness and therapy responses accompanying the ADT-induced metabolic adaptation. We monitored cell viability, clonogenic survival, and adhesive properties, followed by RNA-Seq and biostatistical analyses to pre-assess the molecular mechanisms activated by prolonged ADT that may play a role in HNSCC progression and resistance to therapy. The follow-up validation analyses revealed that repetitive cycles of ADT result in the elevated expression of genes associated with not only metabolic adaptation but also epithelial–mesenchymal transition (EMT), adhesion, pro-survival, and stress response signaling, as well as the activation of the homologous recombination-mediated DNA double-strand break repair pathway.

Overall, our report provides the first evidence that extended ADT can indeed lead to the development of more aggressive and therapy-resistant HNSCC phenotypes. This observation, in conjunction with the contrasting effects of acute ADT, may be relevant for future clinical trial planning.

## 2. Materials and Methods

### 2.1. Expression and Purification of Secreted Recombinant Human Arginase Type 1 (rhARG1) in Yeast Ogataea Polymorpha

The strain-producer of rhARG1 in the methylotrophic yeast *O. polymorpha* was constructed at the Department of Cell Signaling (Institute of Cell Biology, NAS of Ukraine, Lviv, Ukraine) utilizing vector harboring the human *ARG1* gene tagged with C-terminal (His)6 sequence and N-terminally with *Saccharomyces cerevisiae* MF-α leader sequence for efficient secretion (more details of the yeast producer construction and cultivation protocol optimization for rhARG1 will be described elsewhere). Briefly, the recombinant protein was purified by concentrating culture medium on a Vivaflow 50 system (Sartorius, Göttingen, Germany), affinity purified with Ni-NTA agarose (Qiagen, Hilden, Germany), and eluted by a step gradient of imidazole. At each stage of purification, the fractions were monitored for protein concentration [12] and arginase activity and analyzed by SDS-PAGE. The fractions with maximal arginase activity were pooled, dialyzed against phosphate-buffered saline (PBS) supplemented with 10% glycerol, and sterilized by filtration through a membrane with a pore diameter of 0.22 μm. This sample served as one rhARG1 stock for application in our *in vitro* studies. The protein yield was approximately 10–12 mg of arginase per 1 L of culture medium. Depending on the elution fraction, the specific activities of such preparations were 600–1600 µmol min^−1^ mg^−1^ protein.

### 2.2. Assay of Arginase Activity

The activity of rhARG1 as a resulting urea concentration was assessed spectrophotometrically at 520 nm after adding a color-producing reagent and heating at 100 °C for 10 min, essentially as described before [13]. One unit of arginase activity was defined as the amount of enzyme producing 1 µmole of urea per 1 mL of culture medium in 1 min at 37 °C. The standard urea solution (Simko, Lviv, Ukraine) and PBS were used as positive and negative controls, respectively.

### 2.3. Cell Lines and Culture Conditions

The cell line SAS—human head and neck squamous carcinoma of the tongue (Health Science Research Resources Bank, Osaka, Japan)—was used in this study. All sublines including SAS-R9 (“R” stands for “Resistant” to Arg deprivation) were generated by serial exposure to rhARG1, as described in the main text. Cells were routinely tested free of mycoplasma using a PCR Mycoplasma Kit (AppliChem, Darmstadt, Germany), and the genetic profiles of original and sublines were verified via microsatellite analyses based on multiplex PCR kits, as detailed previously [11]. Cultures were routinely grown from validated frozen stocks for ≥2 to ≤20 passages. SAS and SAS-R cells were cultured in high glucose Dulbecco’s Modified Eagle’s Medium (DMEM) supplemented with 10% heat-inactivated fetal bovine serum (FBS) and 50 µg/mL gentamycin and maintained in a humidified 5% CO_2_ in air atmosphere at 37 °C. Exponentially grown cultures were enzymatically dissociated using 0.05% trypsin/0.02% ethylene diamine tetraacetic acid in PBS to obtain single-cell suspensions for passaging and experimental setups. Cells were kept in the exponential growth phase by subculturing twice per week, splitting them in a 1:5 ratio. All culture media, supplements, solutions, and buffers were purchased from Sigma-Aldrich (St. Louis, MO, USA). Arg deprivation was achieved by administering rhARG1 (obtained as described above in Section 2.1) to the complete DMEM (CM) at a concentration of 2 U/mL or by applying a specific formulated medium DMEM (HyClone Laboratories, Logan, UT, USA) lacking arginine (AFM) supplemented with 10% dialyzed FBS (Sigma-Aldrich, St. Louis, MO, USA) devoid of small molecules including amino acids (<10 kDa cut off). In some experiments, the precursor of Arg—citrulline (Cit)—was added at an Arg-equimolar concentration of 0.4 mM.

### 2.4. Cell Viability Assay

Cell growth dynamics and cell viability were assessed by the trypan blue exclusion test and the MTT assay (with TECAN GENios Plus microplate reader), carried out as described earlier [14]. All experiments were conducted with biological triplicates per plate, and each assay was independently repeated at least three times unless indicated otherwise. Data are presented in % relative to untreated (control) cells at point “zero”.

### 2.5. Wound Healing Assay

Cell migration was evaluated *in vitro* using the classical wound healing assay according to [15]. In brief, scratch wounds of approximately 0.5 mm in width were created in cell loans by a thin pipette tip after their previous exposure to CM or AFM for 48 h, followed by transfer to Arg-rich medium. The migratory area was analyzed using the ImageJ Version 1.54 software (NIH, Maryland, USA), and cell migration was quantified as the proportion of the cell-free wounded area at a defined time point relative to initial wound area (0 h) set to 100%.

### 2.6. Slow Aggregation Assay

In the cell–cell homotypic adhesion assay, cell suspensions were inoculated at concentrations of 5 × 10^5^ cells per well into 6-well agarose-coated plates and incubated for 48 h essentially as described [15]. The formed aggregates (sphere cultures of more than 30 cells) were examined under an inverted phase-contrast microscope Delta Optical IB-100 (Mińsk Mazowiecki, Poland) and photographed and the number per well was calculated by ImageJ Version 1.54 software (NIH, Maryland, USA). All experiments were repeated two times in triplicate readings. Statistical analysis was performed using Student’s *t*-test. Differences in the means were considered significant when the calculated *p* < 0.05.

### 2.7. Collagen I Cell Adhesion Assay

Cell–matrix heterotypic adhesion assay was conducted essentially as described in [15]. SAS and SAS-R9 cell suspensions of 5 × 10^4^ untreated cells were seeded into 96-well plates coated with collagen type I in the concentration of 50 µg/mL. After 2 h or 3 h of incubation at 37 °C, non-adherent cells were removed, and the adherent cells were counted using a hemocytometer. All experiments were repeated three times with three technical replicates. Adhesion efficiency was expressed as the percentage of adherent cells from the total number of cells seeded.

### 2.8. Western Blot Analysis

Western blotting was performed using whole-cell protein extracts from exponentially growing cells, as highlighted previously [11]. Protein content was determined with the Pierce BCA Protein Assay Kit (Thermo Fisher Scientific, Waltham, MA, USA). Proteins were separated on a 10% SDS-PAGE and transferred onto a polyvinylidene difluoride membrane. Various primary antibodies (listed in Supplementary Table S1) were applied according to the manufacturers’ instructions to monitor specific non-phosphorylated and phosphorylated proteins of interest. Horseradish peroxidase-conjugated goat anti-mouse or anti-rabbit IgG (1:2000) secondary antibodies (Cell Signalling Technology, Leiden, The Netherlands) and the enhanced chemiluminescence system SuperSignal™ West Dura (Thermo Scientific, Dreieich, Germany) were used for the detection of immunoreactive proteins. Semi-quantitative densitometric analysis of protein bands was carried out using ImageJ Version 1.54 software. Three independent experiments were performed to prepare the samples for Western blot analysis.

### 2.9. Reverse Transcription PCR (RT-PCR)

Total cellular RNA was isolated from cells using the RNeasy Mini kit (Qiagen, Germany) according to the manufacturer’s protocol as described previously [11]. cDNA was synthesized from each sample using the Verso cDNA Synthesis Kit (Thermo Fisher Scientific, Langenselbold, Germany) from 1 μg of total RNA. PCR was carried out in TC48/96 Thermal Cycler (Cleaver Scientific Ltd., Rugby, UK) using the GoTaq Flexi DNA Polymerase Kit (Promega Corporation, Madison, WI, USA) and specific primers for the analyzed genes (see Supplementary Table S2). PCR products were separated by 2.0% agarose gel electrophoresis, visualized by SYBR Green dye staining (Thermo Fisher Scientific, Langenselbold, Germany). Relative intensity of the bands was quantified with the ImageJ software Version 1.54 (NIH, Maryland, USA) and presented after normalization for the reference *ACTB* gene.

### 2.10. Clonogenic Cell Survival Assay

A radiobiological colony forming assay was conducted as previously described [16]. Depending on the given dose, cells were plated into 6-well plates at a density of 200 cells per well (sham irradiation and 2 Gy); 400 cells per well (4 Gy); 800 cells per well (6 Gy); 1600 cells per well (8 Gy); and 3200 cells per well (10 Gy). After 15–16 h, cells were irradiated with the indicated doses of X-rays delivered by a Yxlon Y.TU 320 (200 kV X-rays, dose rate 1.3 Gy/min at 20 mA) filtered with 0.5 mm Cu. A Duplex dosimeter (PTW, Freiburg, Germany) was used to measure the absorbed dose. After irradiation, cells were incubated for 10 days in a humidified 5% CO_2_, 37 °C incubator. Colonies were then fixed with 10% formaldehyde (VWR International) and stained with 0.05% crystal violet (Sigma-Aldrich). Colonies of more than 50 cells were counted using a stereomicroscope (Zeiss, Oberkochen, Germany). The plating efficiencies (PE) and survival fractions (SFs) were calculated as reported earlier [17].

### 2.11. γH2A.X Staining

Analysis of γH2A.X foci was performed for assessing residual DNA double-strand breaks as we discussed previously [18]. In brief, cells were plated onto 8-well Millicell^®^ EZ chamber slides (Merck Millipore) at a density of 30,000 cells/well. The following day, cells were irradiated with 4 Gy X-rays delivered by a Yxlon Y.TU 320 system (200 kV X-rays, dose rate 1.3 Gy/minute at 20 mA, filtered with 0.5 mm Cu). The absorbed dose was measured with a Duplex dosimeter (PTW, Freiburg, Germany). Cells receiving sham irradiation served as controls. Then, 24 h after irradiation, cells were fixed with 3.7% formaldehyde (Thermo Fisher Scientific) and permeabilized with 0.25% Triton X100 (Sigma-Aldrich, St. Louis, MO, USA). Samples were blocked with 10% BSA (Fisher Scientific) in PBS and incubated with the primary antibody (γH2A.X (Ser139), clone JBW301, Merck, Darmstadt, Germany) diluted at 1:1000 in 10% BSA/PBS at +4 °C overnight. The next day, samples were exposed to the secondary antibody (Alexa Fluor 488, Thermo Fisher Scientific, Dreieich, Germany) diluted at 1:500 in 3% BSA/PBS. Nuclei were stained with DAPI (1 µg/mL DAPI in PBS). The images were taken using a confocal Leica SP5 microscope (Leica Microsystems, Wetzlar, Germany). Images were analyzed using Fiji/ImageJ2 software.

### 2.12. RNA-Seq Analysis

SAS and SAS-R9 were divided into three flasks each and passaged independently 3 times until RNA isolation. Equal quantities of RNA from samples were used to prepare the pooled RNA-seq samples. Genewiz Germany (from Azenta Life Sciences, Leipzig, Germany) produced the RNAseq data, and the row counts matrix was used for further processing. Genes with the number of reads below the noise level were filtered out, and samples were quantile normalized. Next, the data set was log2 transformed and represented as fold change relative to the control sample (SAS). A total of 192 gene sets corresponding to Human RT2 Profiler PCR Arrays were retrieved from the Qiagen website and evaluated for deregulation in SAS-R9 by the Wilcoxon signed rank test. The most significantly deregulated gene sets were further assessed for relevance to functional alterations observed in SAS-R9.

### 2.13. Statistical Analysis

Each experiment was independently performed at least three times unless indicated otherwise, and data are presented as the mean ± S.D. Levels of significant differences between groups were determined by paired or unpaired two-tailed Student’s *t*-tests for unequal variances. Statistical analysis of γH2A.X was performed using an unpaired two-tailed *t*-test. The cell survival curves were analyzed using SPSS v.23 software by linear-quadratic formula S(D)/S(0) = exp(-αD-βD2) using stratified linear regression after transformation by the natural logarithm. The difference was considered to be statistically significant at the level of *p* < 0.05. A significant difference between data was defined as * *p* < 0.05 and ** *p* < 0.01.

## 3. Results

### 3.1. Development and Verification of SAS Sublines upon ADT

To gain insight into the mechanisms involved in the acquired resistance of tumor cells to amino acid restriction therapy, such as enzymatic ADT, we isolated sublines of HNSCC SAS cells “adapted” *in vitro* to Arg deprivation. The SAS cell line was chosen because we found it earlier to be quite sensitive to arginine deprivation in 2D culture compared to other HNSCC cell models using Arg-free media [11]. In the present study, we modeled ADT by utilizing preparations of affinity-purified Arg-hydrolyzing His6-tagged recombinant human arginase type 1 (rhARG1) obtained from a yeast *Ogataea polymorpha* producer (cf. M&M). The respective sterile rhARG1 preparations had a purity of >98% and a specific activity of 1600 µmol min^−1^ mg^−1^ protein, comparable to an earlier reported *Saccharomyces cerevisiae* produced rhARG1 [19]. Notably, we previously demonstrated the effective equivalence of defined Arg-free medium (AFM) and CM + rhARG1 media in terms of an evoked decrease in intracellular Arg levels and initiation of cell cycle arrest in non-HNSCC tumor cell lines [20,21,22]. Hence, we intended to use CM + rhARG1 medium in the present study as a mode of ADT to mimic the *in vivo* enzymotherapeutic situation more closely.

**Figure 1:**
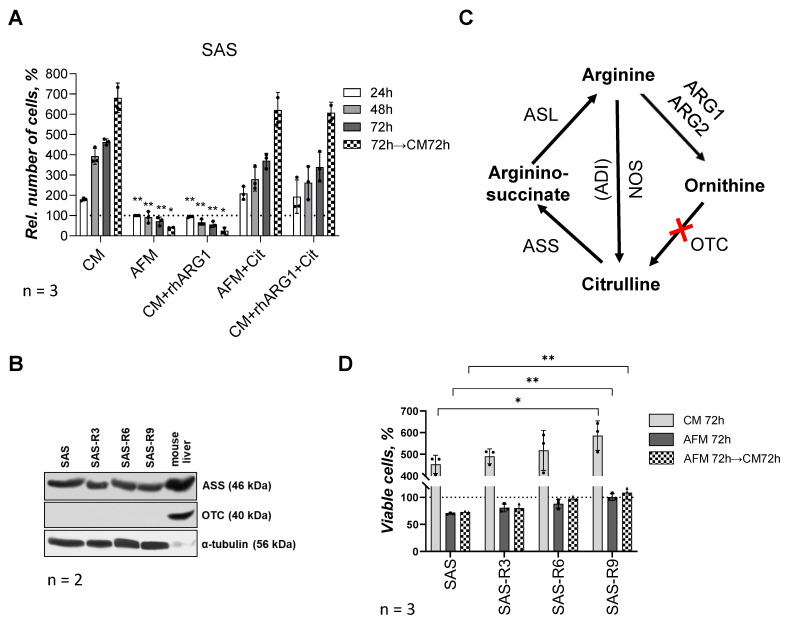
SAS cells and SAS-R sublines differ in their sensitivity to ADT. (**A**) Viability of parental SAS cells under different approaches of Arg starvation and supplementation of Cit in MTT assay: CM—complete medium; AFM—Arg-free medium; rhARG1—recombinant human arginase type 1 (2 U/mL); Cit—citrulline (0.4 mM). Data presented in % of level of untreated (control) cells at “zero” point (means ± standard deviation of N = 3 independent experiments). (**B**) Representative Western blot sets of SAS cells and ADT-adapted sublines SAS-R3, SAS-R6 and SAS-R9 showing expression of ASS and OTC proteins (α-tubulin was used as a sample loading control, N = 2). Murine liver lysate was applied solely as qualitative positive control to confirm presence of ASS and OTC. (**C**) Schematic representation of Arg anabolic and catabolic reactions: ARG1, ARG2—arginase type 1 and 2; OTC—ornithine transcarbamylase; ASS—argininosuccinate synthetase; ASL—argininosuccinate lyase; ADI—arginine deiminase; NOS—nitric oxide synthase; red cross—a lack of enzyme OTC, or it deficiency. (**D**) Viability and re-growth potential of parental and derivative SAS cell sublines after 72 h of Arg deprivation. Number of cells in Trypan Blue test is presented as percentage of levels of untreated corresponding cells at 0 h and expressed as average means ± standard deviation of N = 3 independent experiments; * *p* ≤ 0.05 and ** *p* ≤ 0.01 compared with corresponding SAS cells, as analyzed by Student’s *t*-test. The original Western blot images are provided in the Supplementary Materials.

For verification, we first compared SAS cell viability when cultured in complete DMEM (CM), in formulated AFM, and in Arg-containing complete medium supplemented with 2 U/mL rhARG1 (CM + rhARG1). The data documented in Figure 1A confirm that both methods of selective exogenous Arg withdrawal lead to an analogous growth inhibition in the SAS cell model. SAS cells arrest and eventually die when Arg is withdrawn in the absence of citrulline. Furthermore, and in contrast to most other tumor cell line models studied earlier [21], after 72 h of ADT, SAS cells can no longer recover considerable growth upon Arg re-supplementation (Figure 1A); cf. [11]. Notably, ADT in the presence of excess of the anabolic Arg precursor citrulline (Cit, 0.4 mM) is metabolically less limiting—although the increase in relative cell number over time was still lower compared to the Arg-rich environment. Yet, re-supplementation of Arg, in this case, resulted in full growth recovery (Figure 1A).

In the following, selection of SAS sublines was performed by means of repeated 72 h exposures of SAS cells to Arg-free (CM + rhARG1 for 72 h) followed by Arg-rich (CM) conditions until a relevant proportion of cells recovered. After every three rounds of media changes and exposures to Arg-free conditions, respectively, the regrowing cell population was evaluated for its sensitivity to Arg deprivation relative to the initial SAS cells. The survival and re-growth potential of the initial SAS cells and of the derived sublines, designated as SAS-R3, -R6, and -R9 (where the number corresponds to the sequential exposures to ADT conditions), revealed that SAS-R6 and SAS-R9 sublines exhibit a significantly better survival in Arg-deprived medium relative to SAS and SAS-R3 cells (Figure 1D). The ADT-adapted sublines apparently also exhibited a progressively enhanced (up to 30% in SAS-R9) proliferation in CM (Figure 1D). However, like the original SAS cells, the sublines could not resume growth kinetics after ADT in an Arg-rich but Cit-free environment over 72 h. This finding is in line with the fact that the sublines do not differ from their ancestor concerning (i) the high baseline level of ASS, an enzyme catalyzing the condensation of Cit and aspartate to argininosuccinate, an immediate precursor of Arg (Figure 1B) and (ii) the deficiency in ornithine OTC—an enzyme that converts the arginase degradation product ornithine to Cit (Figure 1B,C). Therefore, SAS cells and their derivates are Arg auxotrophs in a Cit-free *in vitro* environment and their semi-resistance to ADT is not due to the restored Arg anabolism. The isolated sublines were also confirmed to maintain their specific properties after months-long storage in a frozen state. In the following, we comparatively studied the features of the terminal subline SAS-R9, which differed most prominently in survival from the original SAS, in more detail.

### 3.2. SAS-R9 Cells Exhibit Features Indicating Elevated Metastatic Potential

In order to elucidate whether and how prolonged adaptation to ADT affects the malignant potential of HNSCC cells, we examined clonogenic, adhesive, and motile properties of the SAS-R9 subline versus the parental cell line SAS.

**Figure 2:**
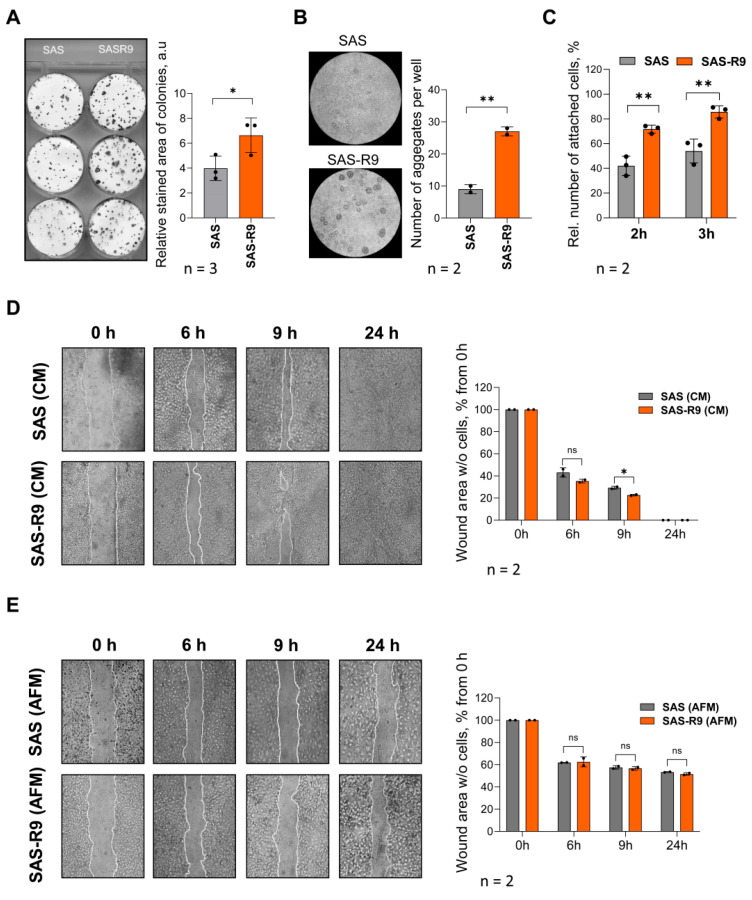
SAS-R9 demonstrates exacerbated clonogenic, adhesion, and migration properties. (**A**) Representative images and calculated relative stained areas of colonies (consisting of at least 50 individual cells) of SAS and SAS-R9 cells. Data represented as means ± standard deviation of N = 3 independent experiments, * *p* ≤ 0.05 compared with SAS cells. (**B**) Representative images and calculated number of aggregates in cell aggregation assay (cell–cell homotypic adhesion). A total of 500,000 cells per well were allowed to form aggregates on agarose-coated 6-well plates for 48 h in CM. Data represented as means ± standard deviation of N = 2 independent experiments, ** *p* ≤ 0.01 compared with SAS cells. (**C**) Percentage of adherent cells in cell–matrix heterotypic adhesion to collagen I assay. Cells were allowed to attach to collagen-coated plates for 2 or 3 h, non-adherent cells were removed, and adherent cells were counted using hemocytometer. Results were expressed as percentage of adherent cells out of total number of cells seeded from N = 3 experiments. (**D**,**E**) SAS and SAS-R9 cell migration in wound healing assay in CM (**D**) and under Arg-free conditions (**E**). Representative phase-contrast images (magnification X40) of cell lawns immediately after scratching (0 h) and 6, 9, and 24 h post-wounding. Cell migration rate into cell-free wound was evaluated by ImageJ software and calculated as percentage of total cell-free scratch area at 0 h. * *p* ≤ 0.05, SAS-R9 vs. SAS cells at indicated time points, Student’s *t*-test. n.s.—not significant. All experiments were performed N = 2 times.

Clonogenic proliferation measured by the relative area of the single-cell-derived colonies revealed that SAS-R9 cells displayed a significantly greater clonogenic proliferation rate than the initial SAS line; depending on the experimental conditions, the plating efficiency increased to >150% of the parental SAS cells (Figure 2A).

We also found an almost three-fold elevated aggregation capacity of SAS-R9 cells using a homotypic cell aggregation assay (Figure 2B). An analysis of cell–matrix heterotypic adhesion revealed that SAS-R9 cells also have a higher ability to adhere to collagen-coated dishes 2–3 h after cell attachment (Figure 2C).

For basic cell motility assessment, we applied the “wound healing assay” using both Arg-free (AFM) and Arg-rich (CM) conditions (Figure 2D,E). The migration rate of SAS-R9 in CM was roughly higher by 10% than of the parental SAS cells (see time points of 6 and 9 h in Figure 2D). Arginine withdrawal profoundly inhibited the ability of both SAS and SAS-R9 cells to migrate into the scratch area, and full wound-closure was not achieved even after 24 h of incubation. However, the inhibition was less pronounced in the SAS-R9 subline (Figure 2E).

In summary, the SAS-R9 subline that underwent multiple exposures to ADT exhibited features tentatively indicating enhanced metastatic properties.

### 3.3. Cell Adaptation to ADT Is Associated with Genome-Wide Transcriptional Changes

**Figure 3:**
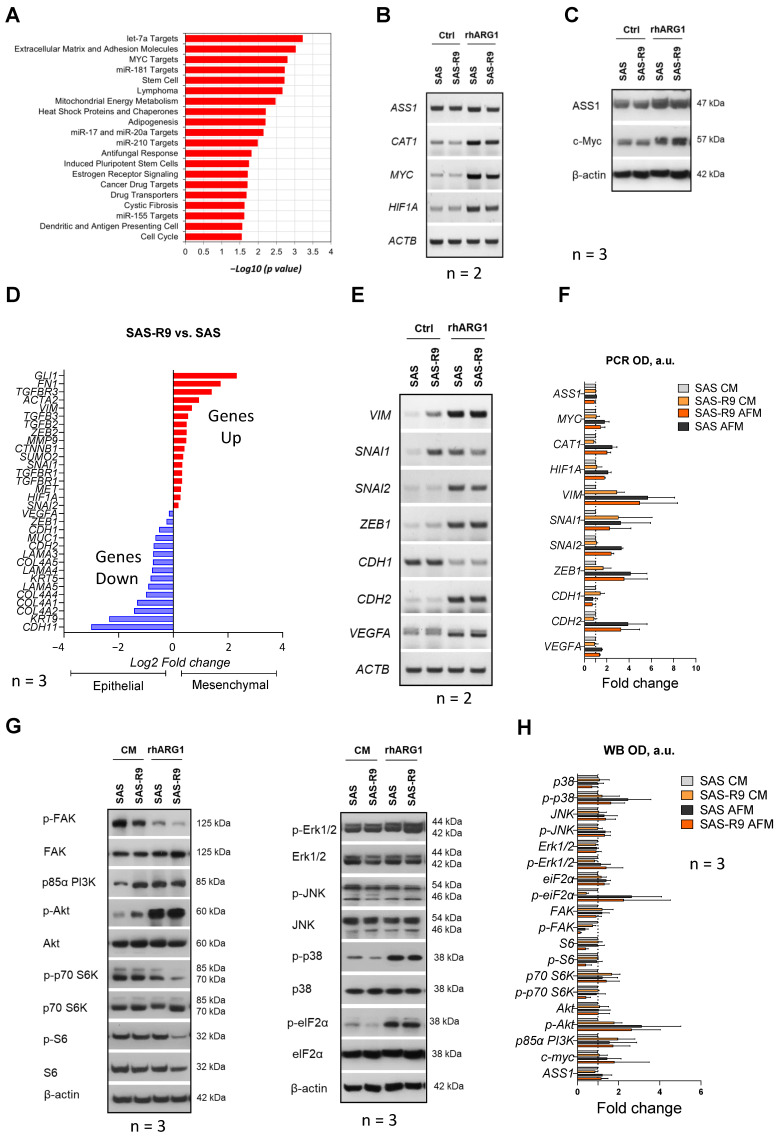
Molecular mechanisms differently regulated in SAS and SAS-R9 cells. (**A**) Gene sets that are most significantly deregulated in SAS-R9 (2-fold change or more) based on GO pathway analysis. The gene lists are provided in Supplementary Figure S1. SAS-R9 cells have an increased EMT phenotype compared to parental SAS cells. (**B**) Representative RT-PCR data sets showing the expression of ASS1, CAT1, MYC and HIF1A genes in two SAS sublines upon 24 h of 2 U/mL rhARG1 treatment (N = 2). (**C**) Representative Western blot data sets showing the expression of ASS1 and c-myc proteins in two SAS sublines upon 24 h of 2 U/mL rhARG1 treatment (N = 3). (**D**) The expression of certain EMT marker genes in SAS-R9 versus SAS cells based on mRNA-Seq analysis. (**E**) Representative RT-PCR data sets and densitometric OD value (**F**) showing the expression of various EMT specific transcription factors and biomarker genes in two SAS sublines upon 24 h of 2 U/mL rhARG1 treatment (N = 2). The *ACTB* gene was used as loading control. (**G**) Representative Western blot data sets showing the expression/activation of proteins of interest from the MAPK and PI3K-PKB/Akt-mTOR signaling pathways in two SAS sublines upon 24 h of 2 U/mL rhARG1 treatment. β-actin was used as loading control (N = 3). (**H**) Densitometric OD value calculated based on Western blot data from (**G**). of N = 3 independent experiments in ImageJ software. The original Western blot images are provided in the Supplementary Materials.

Next, we analyzed and directly compared gene signatures of SAS and SAS-R9 cells by RNA sequencing (RNA-Seq) to identify molecules and mechanisms that might be affected by the cells’ adaptation to ADT. The main pathways and networks modulated in the ADT-adapted cells were identified using Gene Ontology (GO) enrichment analysis based on significantly regulated genes. An analysis of the most significantly up- or downregulated gene sets revealed that SAS-R9 cells have highly activated c-Myc targets (*p* = 1.58 × 10^−3^), mitochondrial energy metabolism (*p* = 3.33 × 10^−3^), and heat shock proteins and chaperones (*p* = 6.15 × 10^−3^), let-7a targets (*p* = 5.96 × 10^−4^), miR-181 targets (*p* = 1.86 × 10^−3^), and miR-17 and miR-20a targets (*p* = 7.10 × 10^−3^) (Figure 3A, Supplementary Figure S1). The pathways most significantly downregulated in SAS-R9 cells included extracellular matrix and adhesion molecules (*p* = 9.35 × 10^−4^), stem cells (*p* = 1.90 × 10^−3^), adipogenesis (*p* = 6.31 × 10^−3^), etc. (Figure 3A, Supplementary Figure S1).

It was previously reported that the acquired resistance to rhARG1 in melanoma cells *in vivo* was due to the altered expression of the two transcription factors c-Myc and HIF-1α, which are positive and negative regulators, respectively, of *ASS1* gene expression [23]. As is consistent with these findings, the c-Myc-driven transcriptional program was highly activated in the SAS-R9 cells, and HIF1α was identified as one of the upregulated miR-17 and miR-20a targets in the SAS-R9 subline (Supplementary Figure S1). Since both transcription factors have a broad range of targets implicated in cell growth, differentiation, apoptosis, and malignant transformation besides the ASS1 [24,25,26,27], we analyzed the expression of these regulators and of some of their targets in our experimental models.

Notably, the amounts of mRNA of the *MYC* and *HIF1*α genes were increased in both SAS and SAS-R9 Arg-deprived cells versus cells cultured in CM (Figure 3B,C). rhARG1 treatment evoked a similar effect on the expression of one of the key transporters of arginine—*CAT1*—and was less pronounced on the *ASS1* gene. However, no evident gene-specific alterations in SAS-R9 versus SAS cells were observed (Figure 3B). We also conducted Western blot analysis of ASS and c-Myc proteins, showing their strict correlation with the gene expression data (Figure 3C). Thus, the observed differences, e.g., elevated proliferation, adhesiveness, and clonogenicity, between SAS-R9 and SAS cells are unlikely to relate to the mechanisms mediated by the transcription factors c-Myc and HIF1α.

### 3.4. SAS-R9 Cells Exhibit Deregulated FAK Signaling and EMT Phenotype

The RNA-Seq data suggest that SAS-R9 cells downregulate a gene set associated with extracellular matrix (ECM) and adhesion molecules (Supplementary Figure S1). As is consistent with this, we observed that SAS-R9 as opposed to SAS cells exhibit lower levels of FAK—a key kinase of integrin-mediated signal transduction pathways associated with cell adhesion, migration, and survival (Figure 3G,H). Integrin-mediated cell adhesion to ECM activates intracellular signaling through phosphorylation of focal adhesion kinase (FAK) [28]. rhARG1 treatment in our setting resulted in a profound decrease in FAK phosphorylation (Tyr397) in both SAS-R9 and SAS cells (Figure 3G,H). Although the expression of many genes involved in the extracellular matrix and cell adhesion is reduced in SAS-R9 compared to the parental SAS cells (Supplementary Figure S1), they exhibited a slightly higher migration potential. This might be explained by the high expression of other key regulators of cell adhesion in this subline, such as *ITGA1, ITGA7, ITGAL,* and *ITGAM*, and ECM proteins, such as *FN1* and *ECM*1 (Figure 3D, Supplementary Figure S1).

To further characterize the differences in the mechanisms governing cell migration in SAS-R9 versus SAS cells, we examined the expression of the key transcription factors and molecules involved in the epithelial-to-mesenchymal transition (EMT) [29]. It was revealed that rhARG1 treatment strongly enhanced the expression of the known EMT genes—*SNAI2*, *ZEB1*, *VIM*, *VEGFA*, and *CDH2*—in both SAS and SAS-R9 cells (Figure 3D–F). At the same time, the expression of the *CDH1* epithelial marker gene significantly decreased under ADT. Notably, in an Arg-rich environment, the SAS-R9 subline exhibited significantly elevated basal expression levels of the EMT reporter genes *VIM, FN1, SNAI1*, etc. (Figure 3D–F). Thus, an altered EMT phenotype in SAS-R9 cells, as compared to the initial SAS cells, accompanies its elevated ADT-resistance.

### 3.5. SAS-R9 Cells Exhibit Altered Regulation of PI3K/Akt/mTOR Signaling

Cell survival and proliferation under metabolic stress are modulated via the key MAPK (mitogen-activated protein kinase) and Phosphatidylinositol 3-kinase (PI3K)/protein kinase B (Akt)/mammalian target of rapamycin (mTOR) signaling pathways [30,31,32,33]. These pathways are transcriptionally regulated by the microRNAs miR-17, miR-20a, and miR-181 [34,35], which appeared to be suppressed in the SAS-R9 subline (Figure 3A, Supplementary Figure S1). However, at the same time, SAS and SAS-R9 cells showed no apparent difference related to MAPK signaling (Figure 3G,H), i.e., comparable phosphorylation levels both under CM or ADT conditions of the main pro-survival MAPK Erk1/2 and JNK (Figure 3G,H), which various stress stimuli can activate [36]. Of note, rhARG1 treatment induced similar profound phosphorylation of stress kinase p38 MAPK in SAS and SAS-R9 cells (Figure 3G), suggesting its important role in sustaining cellular response to Arg deprivation.

The PI3K/Akt/mTOR signaling pathway is crucial for regulating protein translation under amino acid deficiency [37,38]. We revealed an increased expression of a PI3K regulatory subunit p85 and an increased phosphorylation level of Akt Ser475, which is a target of mTOR kinase, in both cell sublines under rhARG1 treatment (Figure 3G). However, the SAS-R9 subline exhibited elevated constitutive levels of both these reporters in CM (expression of p85 PI3K and increased phosphorylation of Akt) relative to the parental cell line (Figure 3G). Also, in SAS-R9 cells, rhARG1 caused a more pronounced inhibition of mTOR activity within 24 h of the treatment, as evidenced by a decrease in the phosphorylation of the mTOR substrates p70S6K and ribosomal protein S6 (Figure 3G). At the same time, no difference was observed between SAS-R9 and SAS cells regarding the activation status of eIF2α (Figure 3G)—another principal regulator of protein translation under amino acid restriction via the GCN2 pathway [39].

Taken together, SAS-R9 cells exhibit an altered regulation of the PI3K/Akt/mTOR pathway, tentatively implicating the protein translation machinery as a mechanism which provides a more efficient pro-survival cellular response to Arg deprivation stress.

### 3.6. ADT Long-Term Surviving Cells Show Activated HR-Mediated DNA Repair Capacity

**Figure 4:**
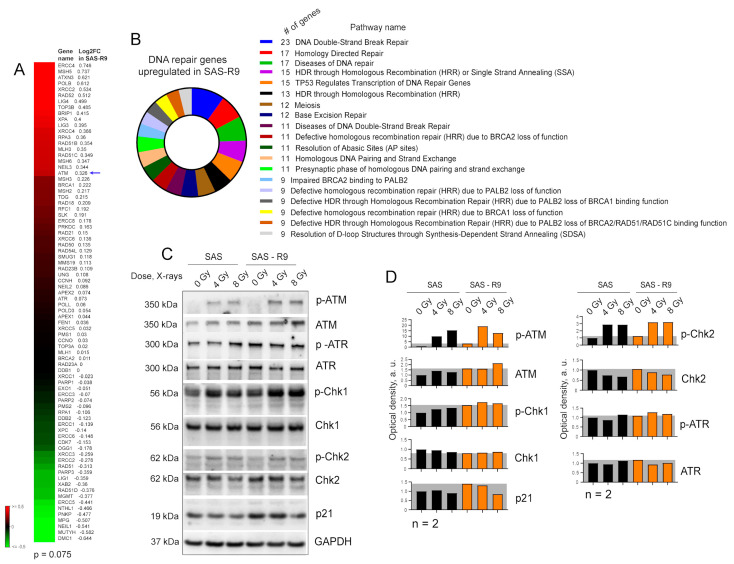
SAS-R9 cells possess the activation of the DNA repair pathways. (**A**) DNA repair gene set shows a strong trend towards upregulation in SAS-R9 (Wilcoxon signed rank test *p*-value = 0.075). (**B**) Reactome pathway analysis [41] of DNA repair genes upregulated in SAS-R9 cells. (**C**) Western blot analysis of DNA damage signaling in SAS and SAS-R9 cells 1 h after irradiation with 4 Gy of X-ray irradiation. Sham-irradiated cells were used as a control. Representative images of one out of two experiments. (**D**) The relative optical density (OD) of C. The ODs of ATM, ATR, Chk1, Chk2, and p21 were normalized relatively to the housekeeping protein GAPDH. The ODs for the phosphorylated proteins p-ATM, p-ATR, p-Chk1, and p-Chk2 were normalized relatively to their non-phosphorylated forms. The original Western blot images are provided in the Supplementary Materials.

RNA-Seq analysis implied that ADT induces the upregulation of genes involved in different DNA repair processes, including DNA double-strand break (DSB) and homology-directed DNA repair (Figure 4A,B). Western blot analysis 1 h after cell irradiation with 4 Gy and 8 Gy of X-rays confirmed high basal level and radiation-induced phosphorylation of ATM and Chk1 in SAS-R9 cells (Figure 4C,D). Pathway analysis revealed that SAS-R9 cells have a high activation of p53-regulated transcription. Consistently, SAS-R9 cells have a higher basal expression level of p21, one of the key p53-regulated target genes [40] (Figure 4C,D).

**Figure 5:**
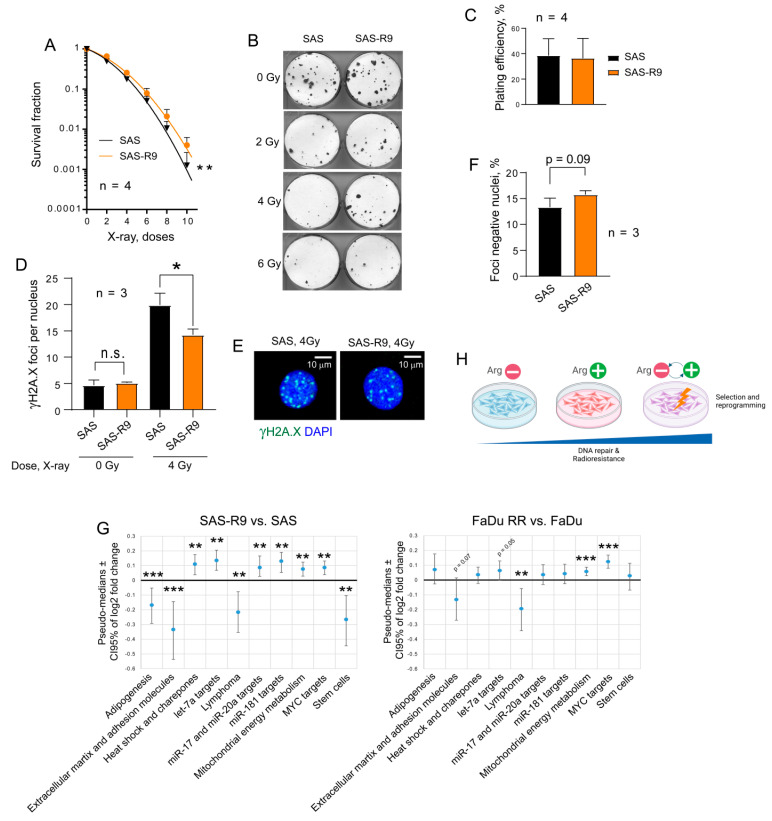
SAS-R9 cells are relatively radioresistant. (**A**) Relative cell radiosensitivity was analyzed using a 2-D radiobiological colony forming assay; error bars = SD; ** *p* < 0.01. (**B**) Representative images of 2-D colonies from one out of four experiments. (**C**) Plating efficacy of sham-irradiated SAS and SAS-R9 cells. Error bars = SD. (**D**) DNA double-stranded breaks (DSBs) were analyzed by γ-H2A.X foci analysis in SAS and SAS-R9 cells 24 h after 4 Gy of X-ray irradiation; * *p* < 0.05. (**E**) Images show the exemplary SAS and SAS-R9 cells with γ-H2A.X foci24 h after 4 Gy of X-ray irradiation. Scale bars = 10 µm; n.s.—not significant. (**F**) The foci-negative populations of SAS and SAS-R9 cells 24 h after 4 Gy of X-ray irradiation. The foci-negative cells were defined as cells with ≤3 foci per nuclei. (**G**) The RT2 transcriptomic gene sets that are most significantly deregulated in SAS-R9 cells have a similar pattern of changes in FaDu RR cells; ** *p* < 0.01; *** *p* < 0.001. (**H**) Our findings suggest that cell selection and reprogramming, which are induced by repeated exposure to ADT, can result in tumor cell resistance to radiotherapy-induced DNA damage.

We next employed a radiobiological clonogenic survival assay to functionally validate the potential activation of DNA repair in SAS-R9 cells (Figure 5A–C). This analysis demonstrated that SAS-R9 cells have acquired relative radioresistance. Likewise, SAS-R9 cells exhibited a significantly decreased number of residual γH2A.X foci per nucleus and have a trend towards a higher number of the γH2A.X foci negative nuclei 24 h after irradiation (Figure 5D–F). These results suggest that repeated exposures and cell adaptation to ADT results in the activation of DNA DSB repair. Although SAS-R9 cells possess a high baseline in DNA damage response signaling activation (Figure 4A–D), SAS-R9 and SAS cells did not differ in the basal levels of DNA DSBs (Figure 5D), suggesting that this activation can be an adaptation to the genotoxic conditions. Indeed, the depletion of Arg might induce a severe deficiency in purine and pyrimidine nucleotides [42] and replication stress [43]. A recent study suggests that Arg shortage leads to tumor cell tolerance to genotoxic stress [43]. Furthermore, a comparative analysis of the transcriptomic profiling in SAS-R9 and HNSCC FaDu RR, which were repeatedly irradiated with 12 fractions of 4 Gy of X-rays and acquired relative radioresistance [44], revealed that the transcriptomic gene sets that are most significantly deregulated in SAS-R9 cells have a similar pattern of changes in FaDu RR cells (Figure 5G). All this evidence suggests that ADT-associated cell selection and reprogramming can result in tumor cell resistance to radiotherapy-induced DNA damage (Figure 5H).

## 4. Discussion

Despite clinically demonstrated therapeutic benefits, acquired resistance to ADT due to upregulated ASS expression remains a major concern for future clinical use [45]. Other factors, including c-myc and stress response pathways, can further contribute to acquired resistance to ADT (reviewed in [46]). The putative development of ADT resistance in primary arginine (Arg)-non-auxotrophic, ASS-positive but ADT-responsive cancer cells, such as HNSCC cells, is also considered. Since these cancer cells may be sensitized to other therapies in an Arg-deprived environment, understanding the mechanisms of acquired resistance is crucial for developing effective combinatorial strategies that can counteract the regulatory adaptations evoked by long-term ADT.

To address this question, we developed an *in vitro* ADT-adapted cell model based on human tongue squamous carcinoma SAS cells, achieved through nine rounds of repeated shifts between rhARG1-treated (Arg-free) and Arg-rich conditions. The established SAS-R9 subline exhibited higher survival following acute ADT, enhanced proliferation, and elevated adhesion, aggregation, and clonogenicity, tentatively indicating increased invasive and metastatic properties. To globally decipher the underlying mechanisms and phenotypes, we performed mRNA-Seq analysis of the whole genome of SAS-R9 versus SAS sublines and confirmed the alterations in the signaling pathway by RT-PCR and Western blotting.

The aggressive and therapy-resistant phenotype of SAS-R9 cells can be attributed to several signaling mechanisms delineated in this study. First, the transcriptional changes in SAS-R9 cells revealed deregulated stress response mechanisms and mitochondria energy metabolism. These data are in line with previously published results, which demonstrate that ADT triggers ER stress response and metabolic adaptation in cancer cells to resolve aberrant protein formation [11].

The Gene Ontology analysis of gene sets deregulated in SAS-R9 compared to the parental counterpart also showed a significant enrichment of several other cancer hallmark gene sets, including c-Myc target genes, heat shock proteins and chaperons, as well as target genes of miR-17, miR-20a, Let-7, and miR-181. Notably, Let-7 miRNA has been demonstrated as a direct regulator of the c-Myc transcription factor [47]. All of the abovementioned miRNAs may play an important role in the phenotypic and functional modifications observed in the ADT-adapted, semi-resistant subline, warranting further analysis.

SAS-R9 cells are characterized by the downregulation of the extracellular matrix and adhesion molecule gene set and elevated expression of key EMT markers (VIM, FN1, SNAI1, etc.). In that context, we also observed a decrease in the activation of FAK, a key kinase of the integrin-mediated signal transduction [48], in the SAS-R9 subline. This alteration could be associated with the changes in cell–cell adhesion and migration seen in these cells. Abundant evidence exists on the involvement of two tightly interconnected MAPK/ERK and PI3K/Akt/mTOR signaling pathways in regulating various hallmarks of cancer cells, like proliferation, stress response, invasion, metastasis, and EMT in different tumor types, including HNSCC [30,32,38,49,50,51,52,53]. As detailed in the results, we did not identify major differences between the SAS and SAS-R9 sublines in the activation status of pro-survival MAPK kinases, ERK1/2, and JNK, except for a slight increase in the phosphorylated form of the ERK1/2 protein in the SAS-R9 cells under acute ADT, which correlated positively with the respective MTT survival data. It is worth mentioning that acute ADT led to a similar and profound activation of the p38 MAPK-mediated pathway in both parental and ADT-adapted derivatives, suggesting its general importance in the response of HNSCC cells to this therapy. The same is true for p-Akt. However, by analyzing the PI3K/Akt/mTOR pathway in more detail, we found that the main differences between SAS and SAS-R9 sublines concern the induction of the p85α subunit of PI3K and p-Akt under Arg-rich conditions and a downregulation of the mTOR substrates p-p70 S6K and p-S6 under acute ADT in the SAS-R9 cells. Notably, the activation of the AKT pathway was reported earlier to contribute to ADT resistance in melanoma cells [23].

ADT, more precisely acute but not extended, sequential ADT, radiosensitizes HNSCC cells by affecting various cellular processes [11]. It induces cell cycle arrest, either in the G1 phase or throughout the entire cell cycle [54], which can both influence how cells respond to radiation. ADT can lead to an accumulation of reactive oxygen species (ROS), thereby enhancing the damage caused by radiation [55]. Cells that are starving in the absence of Arg may, furthermore, exhibit a reduced capacity to repair DNA damage, making them more vulnerable to the effects of radiation.

Contrary to the reported radiosensitizing effect of ADT, a recent study has shown that Arg limitation induces replication stress and confers genotoxic resistance by inhibiting histone H4 translation and promoting proliferating cell nuclear antigen (PCNA) ubiquitination [42]. Additionally, it has been demonstrated that ASS1 metabolically contributes to the nuclear and cytosolic p53-mediated DNA damage response and restrains cell cycle progression by restricting nucleotide synthesis [56]. In line with these findings, SAS-R9 cells demonstrated acquired relative radioresistance and a significantly decreased number of residual γH2A.X foci as a marker of DNA damage. It can be hypothesized that potential deficiency in the nucleotide pool during ADT could make the cells “tuned” to deal with permanent DNA damage. Alternatively, this deficiency can induce the selection of cell populations with activated DNA repair signaling pathways.

Notably, we found a striking similarity between the signaling pathways activated by repeated ADT in SAS-R9 cells and another HNSCC model, FaDu RR, with acquired radioresistance. Our observations suggest that these two types of cancer therapies, when applied repeatedly, could select tumor cells with similar phenotypes. The analysis of the potential difference in sensitivity to ADT in HNSCC with acquired radioresistance should be a focus of follow-up studies.

In summary, our study suggests that repeated, long-term arginine deprivation therapy results in the acquisition of an aggressive and multi-therapy-resistant tumor phenotype in HNSCC cells *in vitro*. These observations shall be verified in additional cell and tumor models *in vitro* and *in vivo* for consideration in future clinical trials.

## 5. Conclusions

In conclusion, the comparative characterization of the ADT-adapted HNSCC SAS-R9 subline revealed a plethora of phenotypic and functional alterations, including more rapid clonogenic growth, increased adhesive properties, elevated ability to undergo EMT, as well as altered DNA repair and radioresistance. This data may raise concerns about the suitability of prolonged ADT as a mono-treatment, but also points in favor of designing new, rational, combined, shorter-term treatments based on ADT, which may also involve radiotherapy for HNSCC.

## Data Availability

The original contributions presented in this study are included in the article/Supplementary Material.

## Supplementary Materials

The following supporting information can be downloaded at: https://www.mdpi.com/article/10.3390/biom15060900/s1. Figure S1: RT2 gene sets that are most significantly upregulated in SAS-R9; Table S1: List of antibodies used in study; Table S2: Primers used for the detection and quantification of various genes by RT-PCR analysis. The original Western blot images are in the supplementary materials.

## Abbreviations

The following abbreviations are used in this manuscript:

ADT	Arginine deprivation therapy
Arg	Arginine
rhARG1	Recombinant human arginase type 1
CM	Complete medium
AFM	Arginine-free medium
Cit	Citrulline
EMT	Epithelial–mesenchymal transition
HNSCC	Head and neck squamous cell carcinoma
ASS	Argininosuccinate synthetase
ERK	Extracellular signal-regulated kinases
mTOR	Mammalian target of rapamycin
PCNA	Proliferating cell nuclear antigen
RT–PCR	Reverse transcription polymerase chain reaction
MAPK	Mitogen-activated protein kinase
eIF2α	Eukaryotic translation initiation factor 2α
FAK	Focal adhesion kinase
Akt	Protein kinase B
PI3K	Phosphatidylinositol 3-kinase
