# Supplementary material for: Adaptation to Arginine Deprivation Leads to a More Aggressive, Therapy-Resistant Phenotype in HNSCC Cells
Source: Biomolecules. 2025 Jun 19;15(6):900. doi: 10.3390/biom15060900 (PMC12190576; doi:10.3390/biom15060900)

Supplementary Table 1. List of antibodies used in the study

Antibody	Source	Cat. Number
Mouse monoclonal antibody anti-AKT	Cell Signaling Technology	# 2920S
Rabbit monoclonal antibody anti-Phospho-AKT (Ser475)	Cell Signaling Technology	# 4060S
Rabbit monoclonal antibody anti-Phospho-p44/42 MAPK (Erk1/2) (Thr202/Tyr204)	Cell Signaling Technology	# 4370S
Rabbit polyclonal antibody anti-p44/42 MAPK (Erk1/2)	Cell Signaling Technology	# 9102S
Rabbit monoclonal antibody anti-Phospho-eIF2alpha (Ser51)	Cell Signaling Technology	# 3597S
Rabbit monoclonal antibody anti-eIF2alpha	Cell Signaling Technology	#5324S
Rabbit monoclonal antibody anti-Phospho-SAPK/JNK (Thr183/Tyr185)	Cell Signaling Technology	#4668S
Rabbit polyclonal antibody anti-SAPK/JNK	Cell Signaling Technology	#9252S
Rabbit monoclonal antibody anti-Phospho-p38 MAPK (Thr180/Tyr182)	Cell Signaling Technology	#4511S
Rabbit monoclonal antibody anti-p38 MAPK	Cell Signaling Technology	#8690S
Rabbit monoclonal antibody anti-Phospho-FAK (Tyr397)	Cell Signaling Technology	#8556S
Rabbit polyclonal antibody anti-FAK	Cell Signaling Technology	#3285S
Rabbit monoclonal antibody anti- Phospho-p70 S6 Kinase (Thr389)	Cell Signaling Technology	#9234S
Rabbit monoclonal antibody anti-p70 S6 Kinase	Cell Signaling Technology	#2708S
Rabbit monoclonal antibody anti-Phospho-S6 Ribosomal Protein (Ser235/236)	Cell Signaling Technology	#4858S
Rabbit monoclonal antibody anti-S6 Ribosomal Protein	Cell Signaling Technology	#2217S
Mouse monoclonal antibody anti-PI3 Kinase p85α	Cell Signaling Technology	#13666S
Rabbit monoclonal antibody anti-c-Myc	Cell Signaling Technology	#5605S
Mouse monoclonal anti-β-Actin	Sigma-Aldrich	A5441
Mouse monoclonal antibody anti-ASS1	Sigma-Aldrich	MABN704
Rabbit polyclonal antibody anti-OTC	Atlas Antibodies	HPA000243
Rabbit monoclonal antibody anti-ATR	Cell Signaling Technology	#13934
Rabbit polyclonal antibody anti-Phospho ATR (Ser428)	Cell Signaling Technology	#2853
Rabbit monoclonal antibody anti-ATM	Cell Signaling Technology	#2873
Rabbit polyclonal antibody anti-Phospho ATM (Ser1981)	Cell Signaling Technology	#5883
Mouse monoclonal antibody anti-Chk1	Cell Signaling Technology	#2360
Rabbit polyclonal antibody anti-Phospho Chk1 (Ser296)	Cell Signaling Technology	#2349
Rabbit polyclonal antibody anti-Chk2	Cell Signaling Technology	#6334
Rabbit monoclonal antibody anti-Phospho Chk2 (Thr68)	Cell Signaling Technology	#2197
Rabbit monoclonal antibody anti-p21	Cell Signaling Technology	#2947
Rabbit polyclonal antibody anti-GAPDH	Santa Cruz Biotechnology	sc-25778

Supplementary Table 2. Primers used for the detection and quantification of various genes by RT-PCR analysis

Target gene	Primer sequence	Product size
ACTB	Forward - CACCCTGAAGTACCCCATCG	199 bp
	Reverse - GCTGGGGTGTTGAAGGTCTC	
ASS1	Forward - GAGCTCTTCATGTACCTGAACG	196 bp
	Reverse - CCAGGCCTTGTTTGATTTTGC	
CAT1	Forward - CCT ACA TCA TCG GTA CTT CAA GCG	207 bp
	Reverse - CCA TGG CCG ACT CTT TCA CAC	
CDH1	Forward - GGC CTG AAG TGA CTC GTA ACG	212 bp
	Reverse - GT TCA GGG AGC TCA GAC TAG	
CDH2	Forward - CAG AAT CAG TGG CGG AGA TC	268 bp
	Reverse - CCT TCT da TGG CGA ATG ATC	
HIF1A	Forward - TGCTCATCAGTTGCCACTTC	180 bp
	Reverse - AAAACCATCCAAGGCTTTCA	
MYC	Forward - GGATTCTCTGCTCTCCTCGAC	220 bp
	Reverse - GCTGTGAGGAGGTTTGCTG	
SNAI1	Forward - GAAAGGCCTTCAACTGCAAA	249 bp
	Reverse - TGACATCTGAGTGGGTCTGG	
SNAI2	Forward - TCG GAC CCA CAC ATT ACC TT	159 bp
	Reverse - TGA GCC CTC AGA TTT GAC CT	
VEGFA	Forward - TGA GCT TCC TAC AGC ACA AC	210 bp
	Reverse - TCG GCT TGT CAC ATC TGC AA	
VIM	Forward - CAG GCT CAG ATT CAG GAA CAG	191 bp
	Reverse - GGC GTC ATT GTT CCG GTT GG	
ZEB1	Forward - GATTCTACACCGCCCAAAAA	245 bp
	Reverse – AAGCGCTTTCCACATTTGTC	

Supplementary Figure 1

RT2 genesets that are most significantly upregulated in SAS-R9 (p-values calculated by Wilcoxon signed rank test)

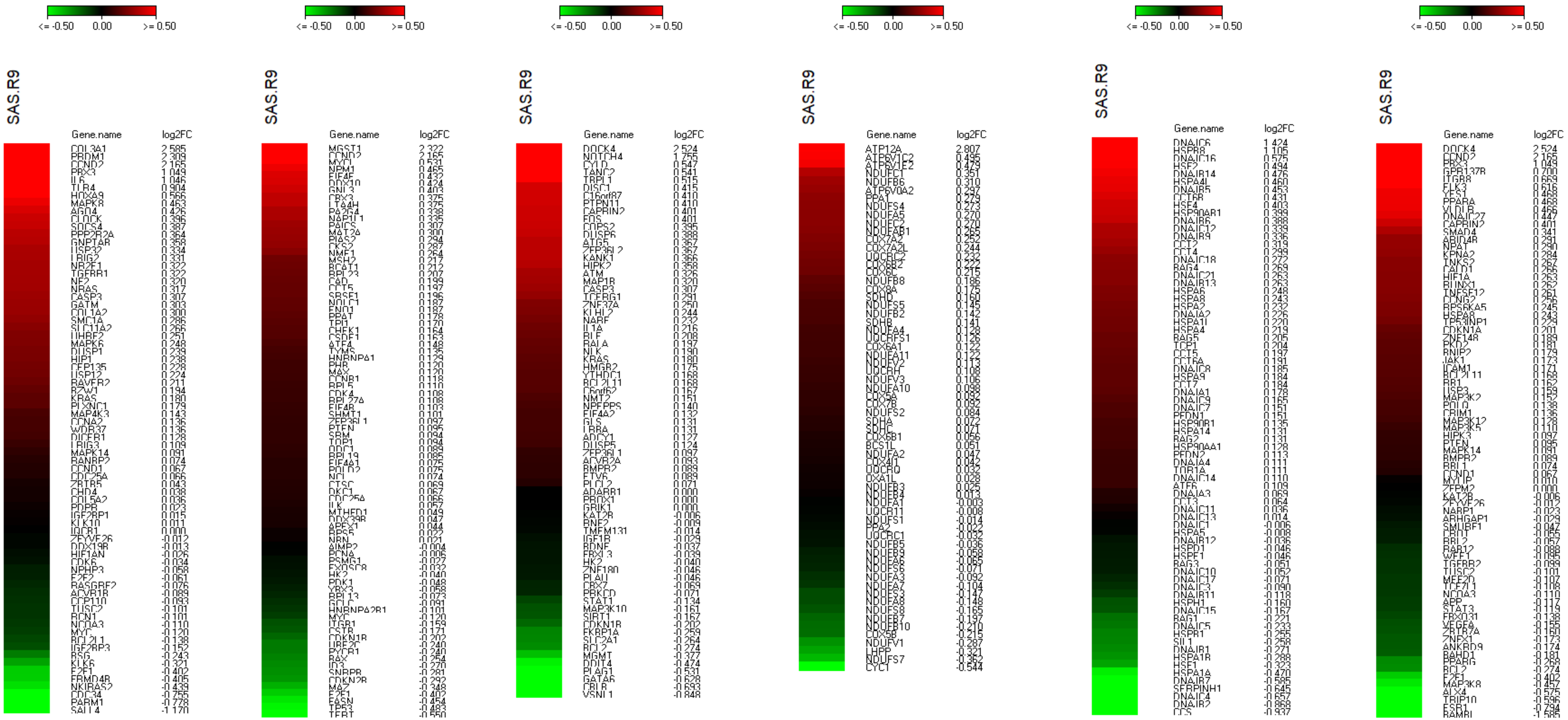

let-7a Targets
p=5.96E-04

MYC Targets
p=1.58E-03

miR-181 Targets
p=1.86E-03

Mitochondrial Energy Metabolism
p=3.33E-03

Heat Shock Proteins and Chaperones
p=6.15E-03

miR-17 and miR-20a Targets
p=7.10E-03

Supplementary Figure 1

RT2 genesets that are most significantly downregulated in SAS-R9 (p-values calculated by Wilcoxon signed rank test)

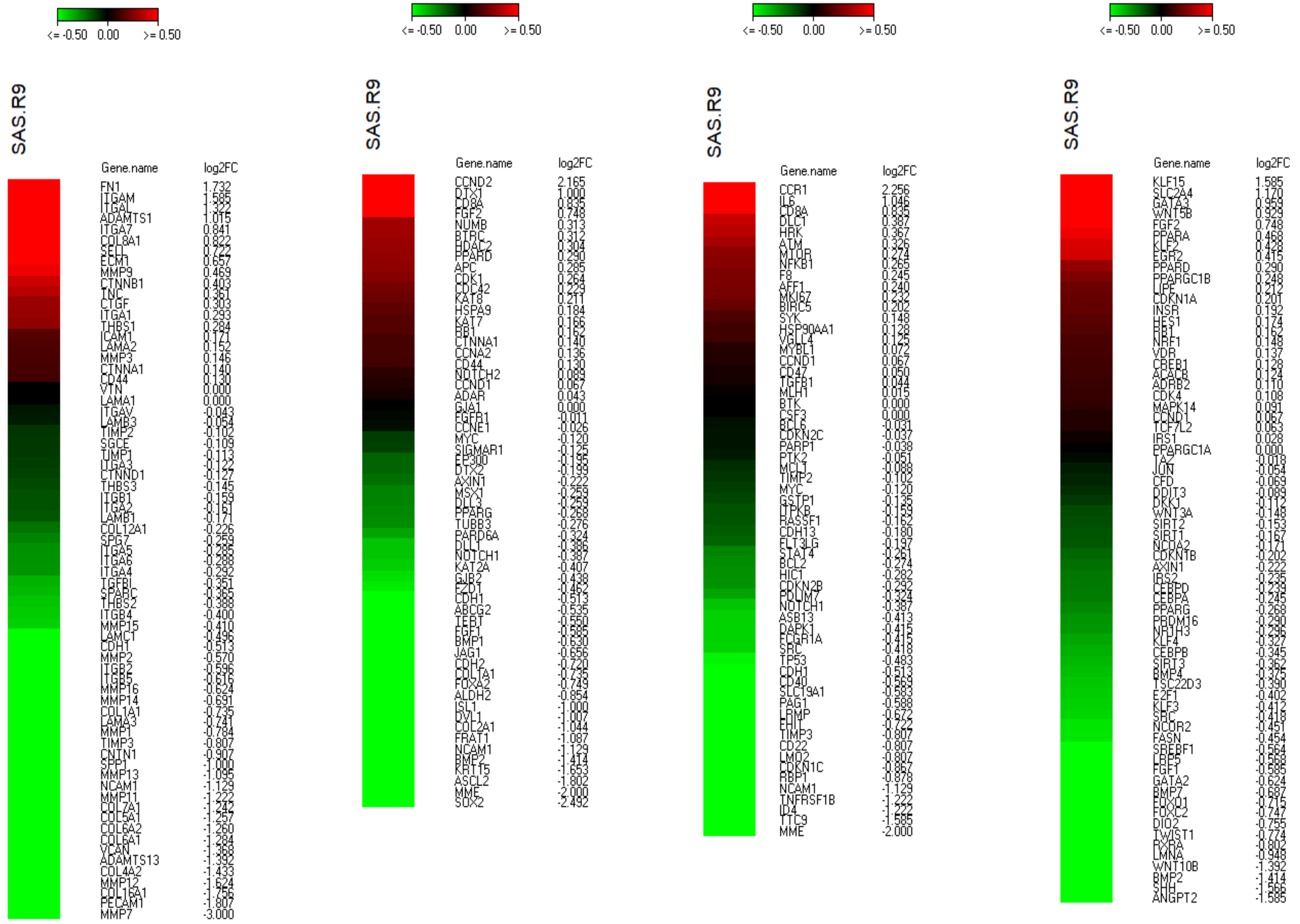

Extracellular Matrix and Adhesion Molecules p=9.35E-04

Stem Cell p=1.90E-03

Lymphoma p=2.14E-03

Adipogenesis p=6.31E-03

Original Western blot images
Figure 2B

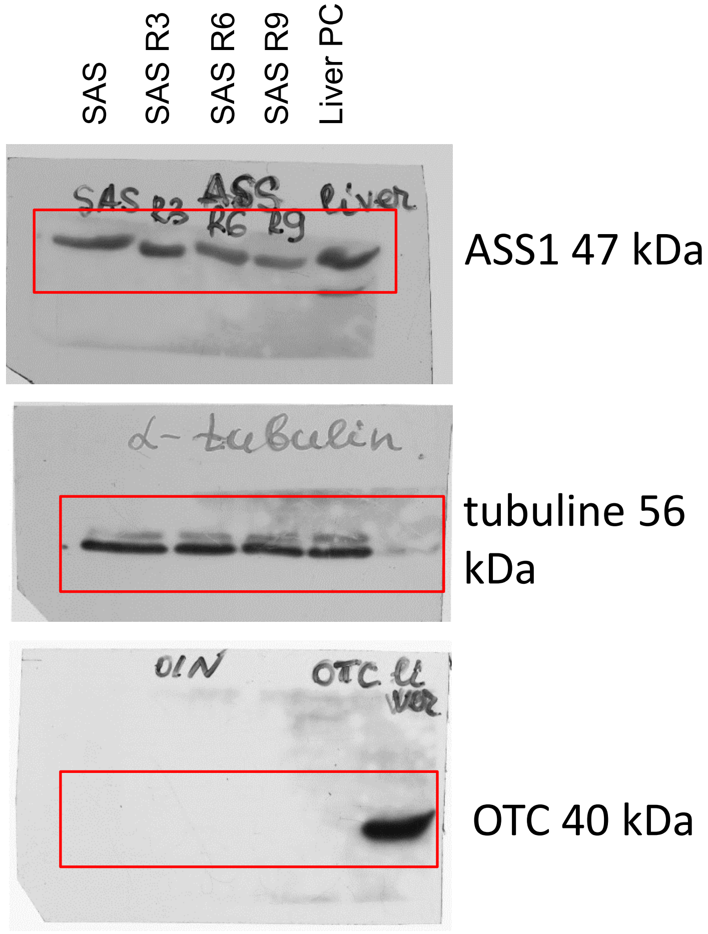

Original Western blot images
Figure 3C

#1,#2, #3 – three independently collected lysates

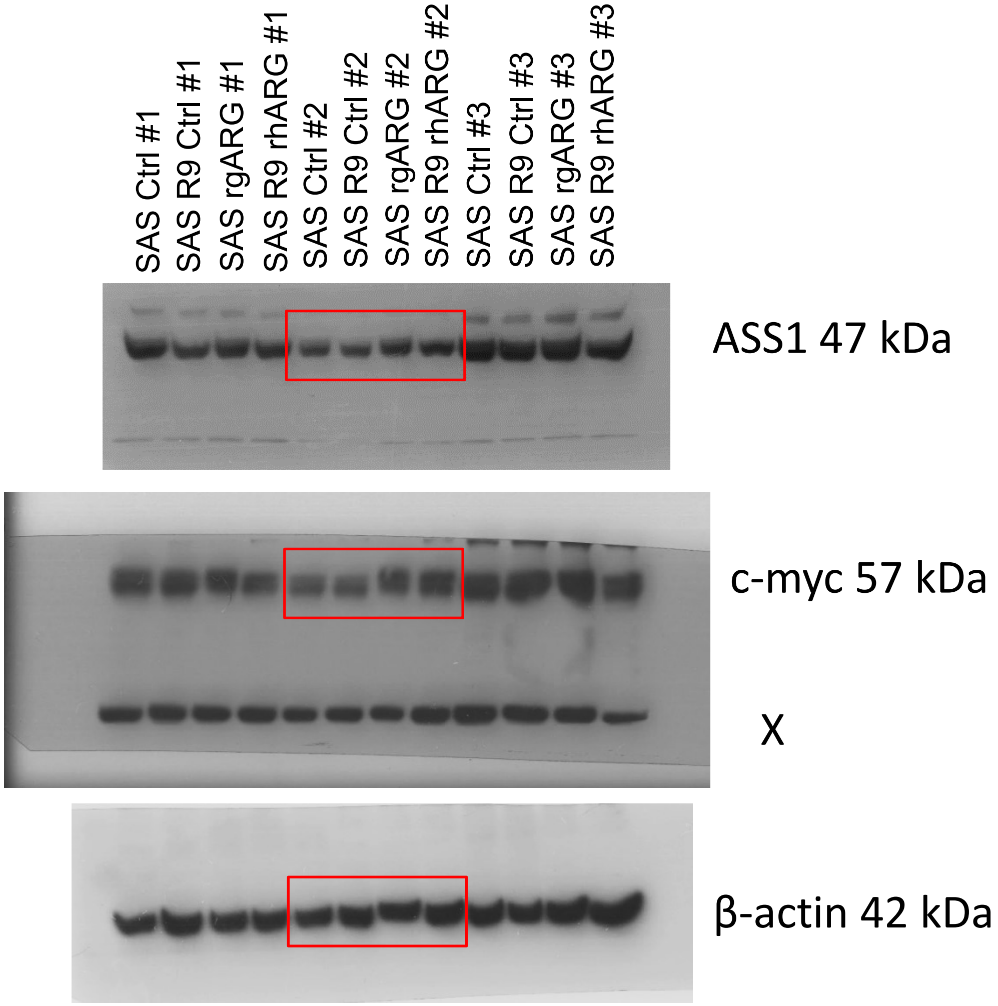

Original Western blot images

Figure 3G

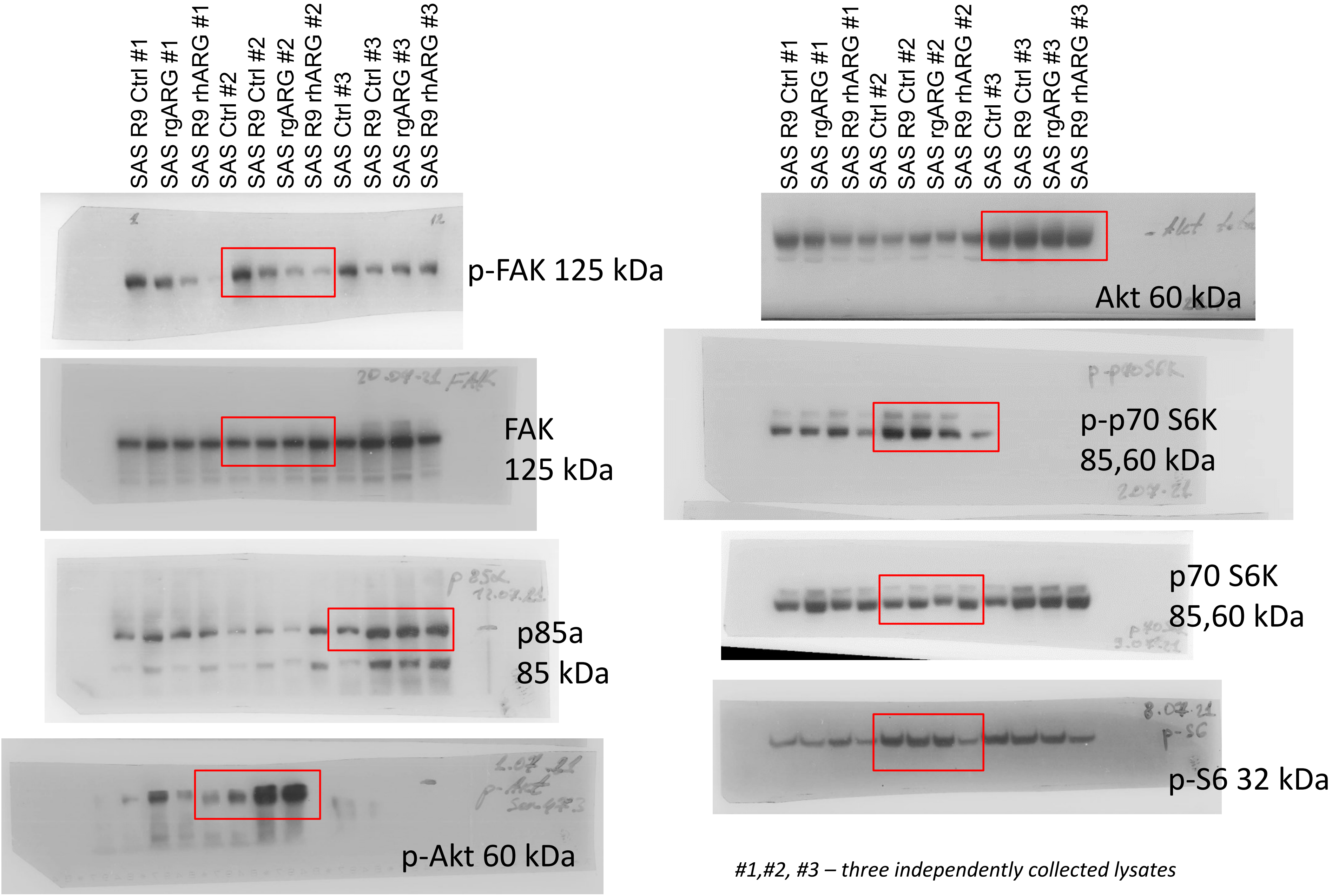

#1,#2, #3 – three independently collected lysates

Original Western blot images

Figure 3G

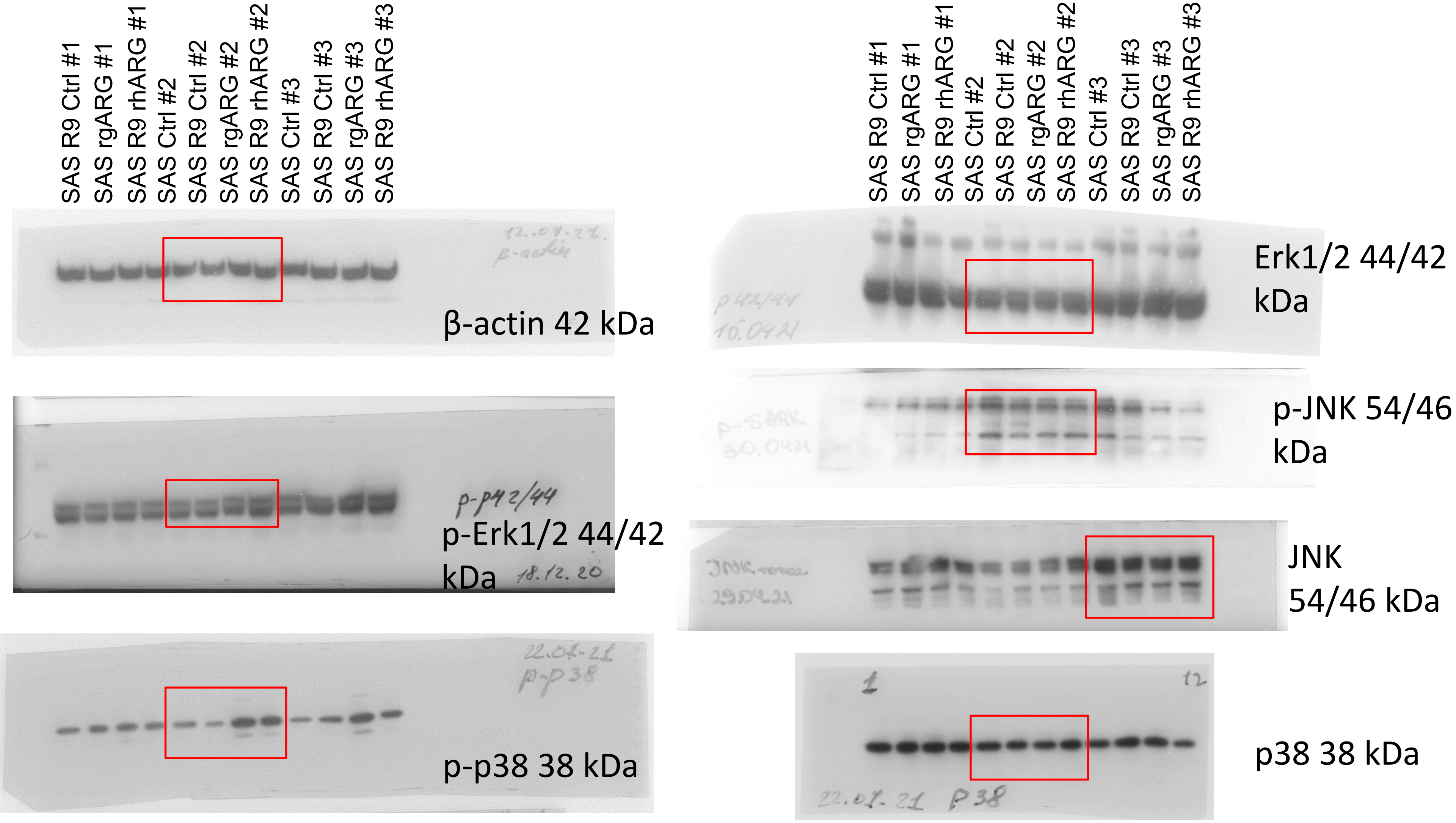

#1,#2, #3 – three independently collected lysates

Original Western blot images
Figure 3G

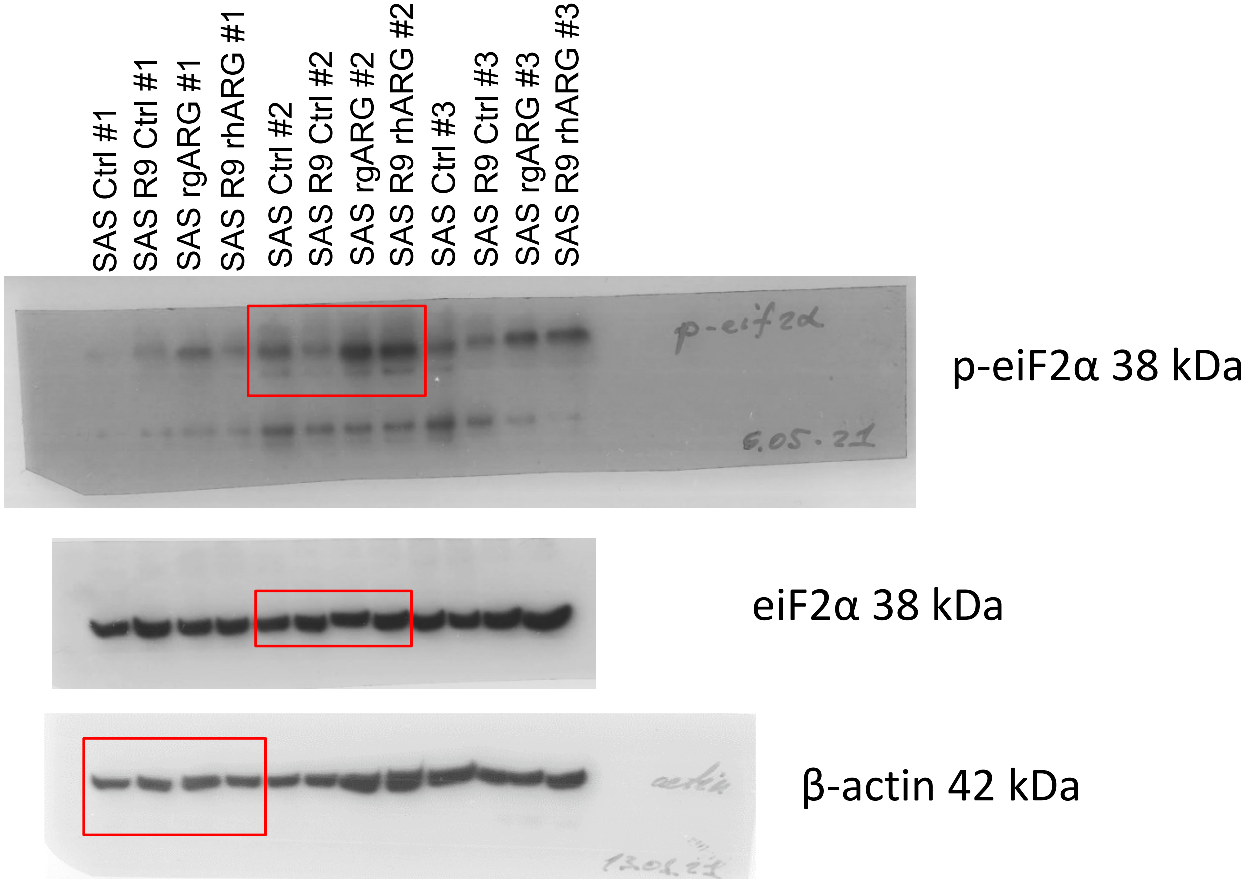

#1,#2, #3 – three independently collected lysates

Original PCR images
Figure 3E

#1,#2 – independently collected lysates

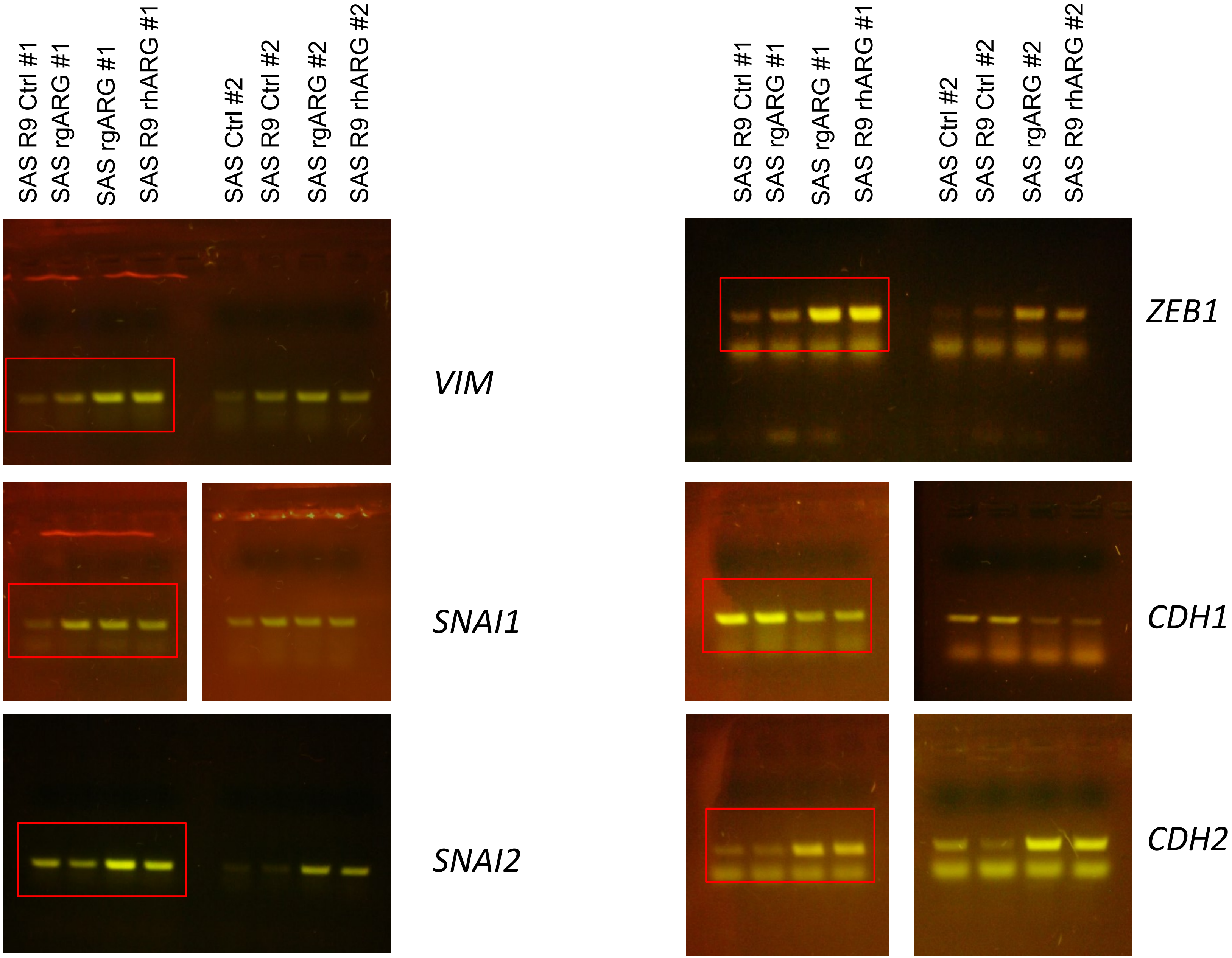

Original PCR images

Figure 3B

#1,#2 – independently collected lysates

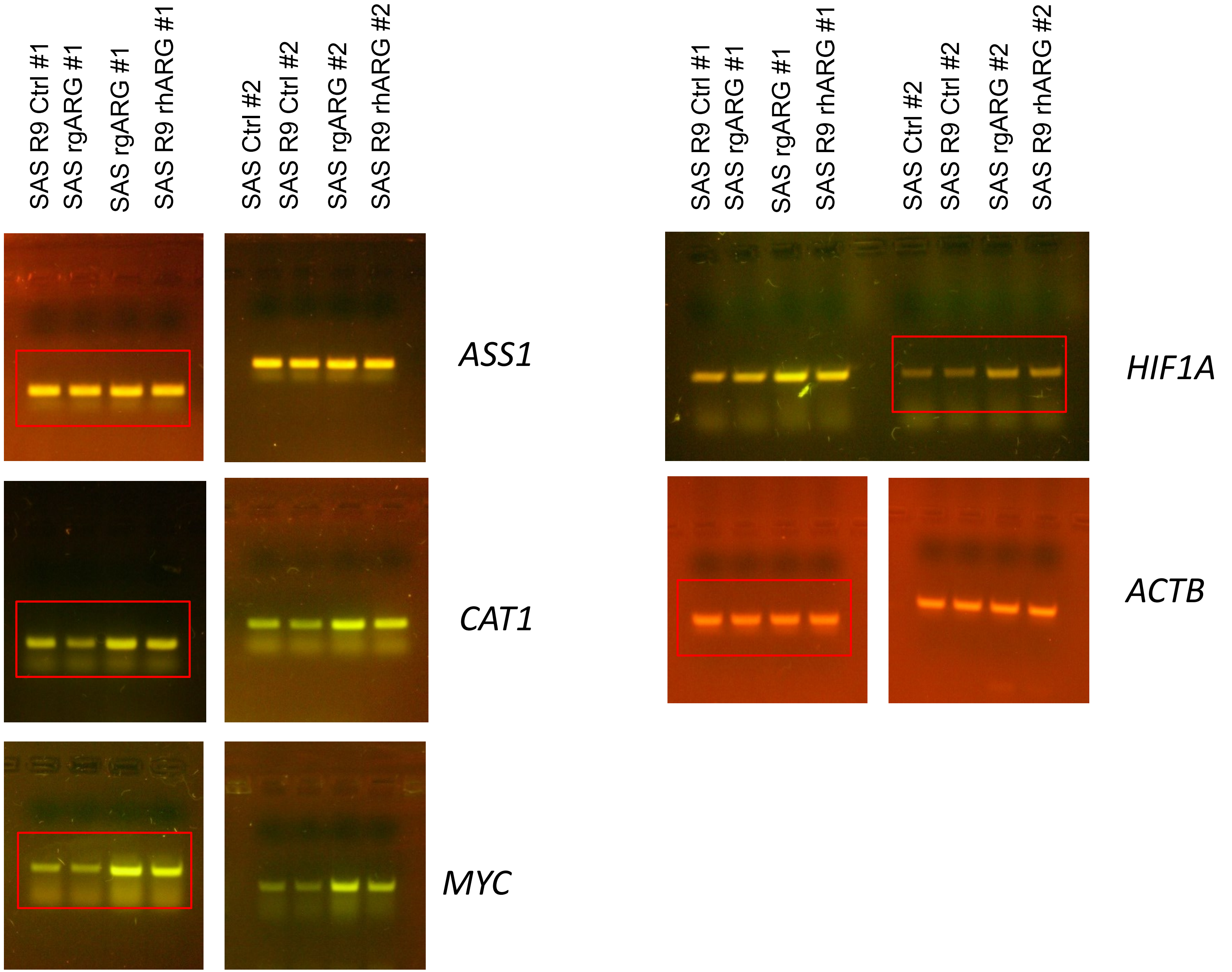

Original PCR images *#1,#2 – independently collected lysates*

Figure 3E

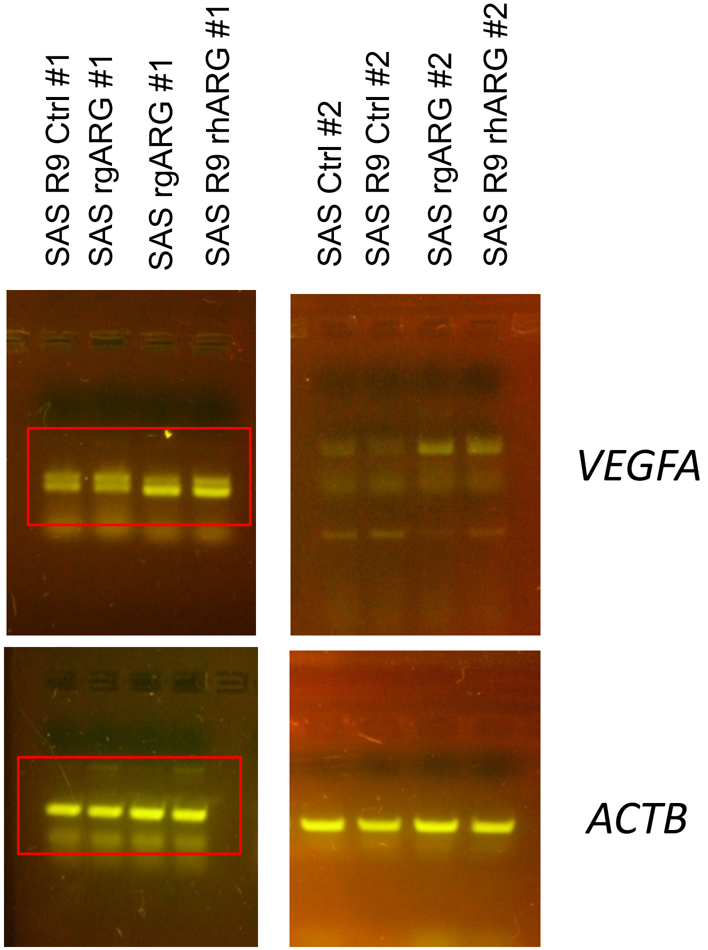

Original Western blot images
Figure 4C

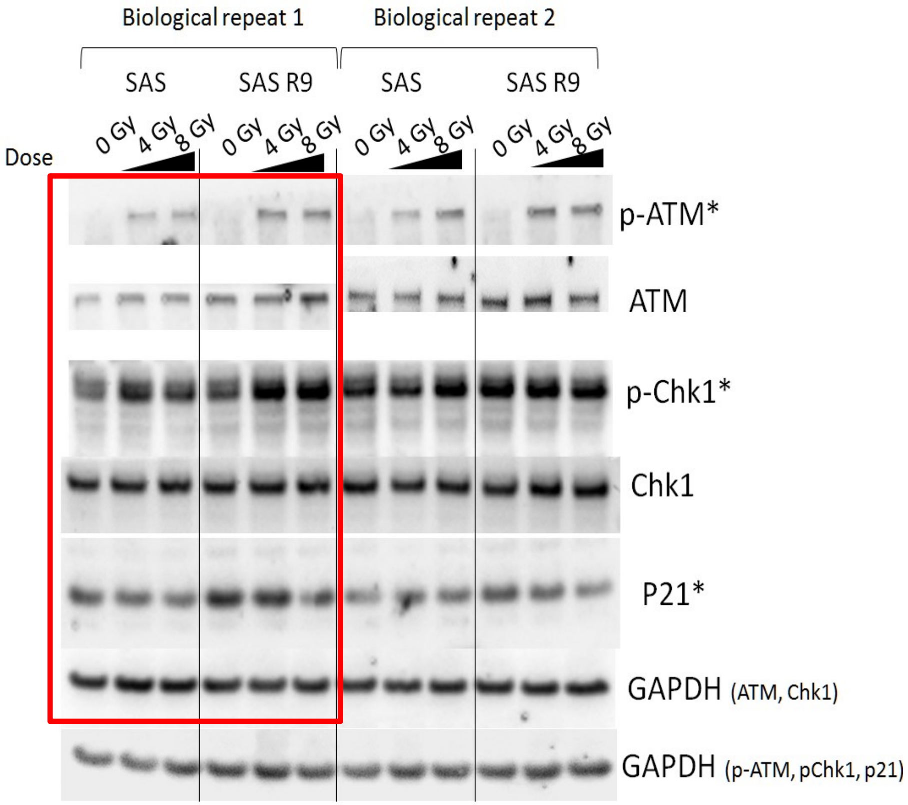

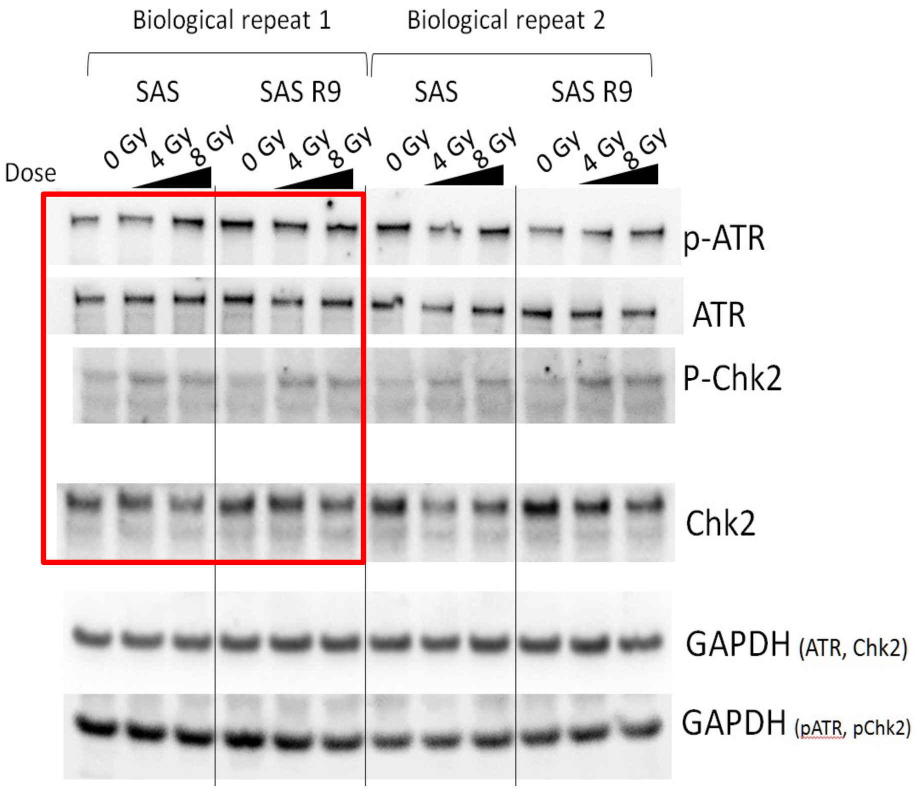

Original Fluorescence microscopy images
Figure 5E

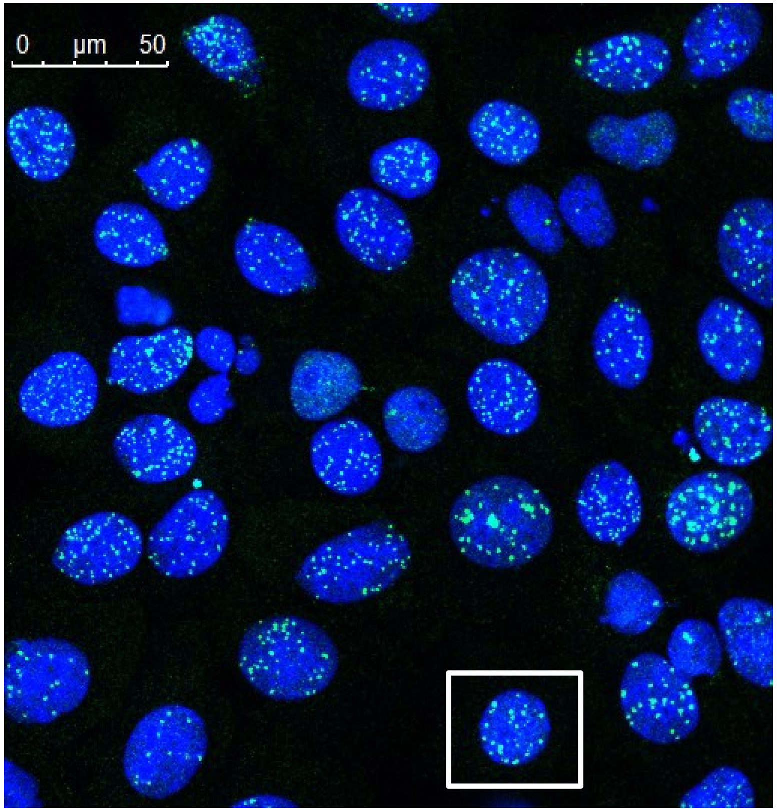

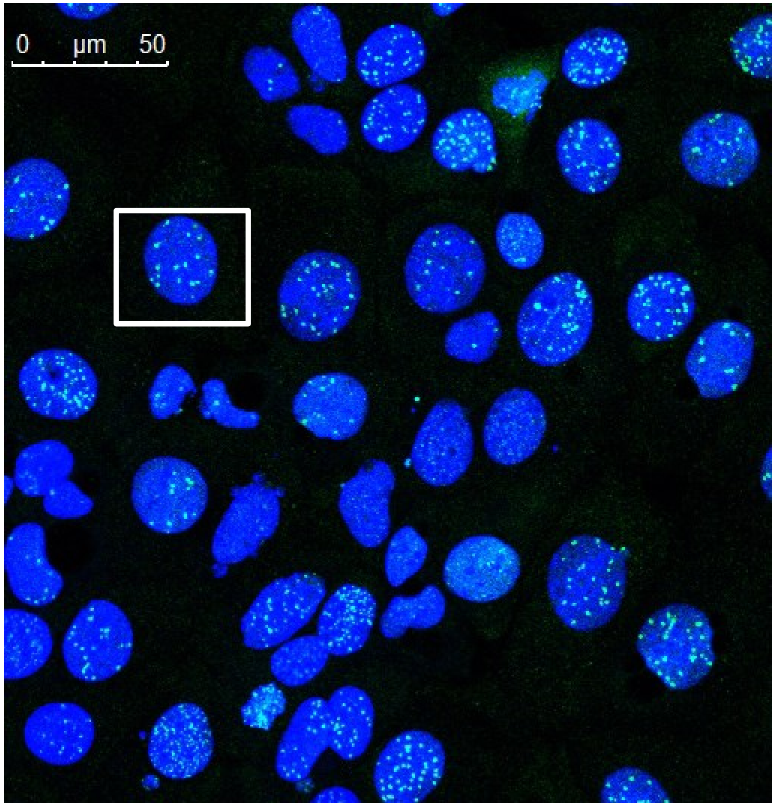

Supplement: Supplementary file 1 [file biomolecules-15-00900-s001.zip › biomolecules-3640790-supplementary.pdf]
